# Trends in the incidence and outcomes of bicycle-related injury in the emergency department: A nationwide population-based study in South Korea, 2012-2014

**DOI:** 10.1371/journal.pone.0181362

**Published:** 2017-07-13

**Authors:** Youn-Jung Kim, Dong-Woo Seo, Jae-Ho Lee, Yoon-Seon Lee, Bum-Jin Oh, Kyoung-Soo Lim, Won Young Kim

**Affiliations:** Department of Emergency Medicine, University of Ulsan College of Medicine, Asan Medical Center, Seoul, Korea; University of New South Wales, AUSTRALIA

## Abstract

**Objective:**

This study aimed to examine trends in the incidence and outcomes of bicycle-related injuries in emergency departments (ED) in South Korea.

**Methods:**

We analysed data from the National Emergency Department Information System database for adult patients (≥20 years) with bicycle-related injuries presenting to EDs in South Korea between January 2012 and December 2014. Riders and bicycle passengers whose injuries were associated with bicycle use were included. Serious outcomes were defined as death at the ED, need for emergency operation, or intensive care unit admission.

**Results:**

The number of people who commute to work by bicycle increased by 36% from 205,100 in 2005 to 279,544 in 2015. Of 529,278 traffic-related trauma cases, 58,352 (11.0%) were bicycle-related, which increased from 7,894 (10.2%) in the first half of 2012 to 12,882 (12.2%) in the second half of 2014 (p < 0.001). However, the proportion of serious outcomes decreased from 5.0% to 4.2% during the study period (p < 0.001). Serious outcomes were most frequent in the elderly (65–74 years) and older elderly (≥75 years) groups and decreased for all but the elderly age group from 10.3% to 9.8% (p = 0.204). The helmet use rate increased from 14.2% to 20.3% (p < 0.001) but was the lowest in the older elderly group (3.6%) without change during the study period (from 4.7% to 3.7%, p = 0.656). A lack of helmet use was significantly associated with serious outcomes (odds ratio, 1.811; 95% confidence interval, 1.576–2.082).

**Conclusions:**

Although the incidence of bicycle-related injuries increased, the proportion of serious outcomes decreased, possibly due to increased helmet use. Public education on safety equipment use is required, especially in elderly populations.

## Introduction

Bicycle riding is a popular leisure activity worldwide. In South Korea, the government has promoted a bicycle-friendly infrastructure since 2010 to benefit the environment and ease urban traffic congestion. The number and length of bike lanes in South Korea have increased from 5,392 lanes and 13,037 km in 2010 to 9,374 lanes and 19,717 km in 2014 [1, 2]. Although detailed demographic data of the typical cycling population in South Korea is not available, according to a population census conducted by the National Statistical Office (NSO), Statistics Korea, the number of people who commute to work by bicycle increased by 36% from 205,100 in 2005 to 279,544 in 2015 [3, 4]. Annual bicycle sales also increased by 40% between 2008 and 2012. As bicycle riding increased in Korea, the incidence of bicycle-related injuries has also increased rapidly.

Although many previous studies have reported the national epidemiology of bicycle-related injuries, most were conducted in countries with well-developed bicycle transportation systems [5–8]. A recent Australian cohort study of adult cyclists estimated 0.29 crashes per 1,000 km cycled (95% CI: 0.26–0.32), with cyclists seeking medical treatment in 8% of these crashes [9]. Head injuries, in particular, are an important fraction of cycling-related injuries [10]. A clear understanding of the current epidemiology of bicycle-related injuries will aid the design and implementation of injury prevention strategies. Thus, the aim of this population-based study was to evaluate trends in the incidence and outcomes of bicycle-related injuries presenting to EDs in South Korea using a nationwide prospective database system.

## Methods

### Study design and population

This nationwide cross-sectional study used prospectively collected data from the National Emergency Department Information System (NEDIS) collected between 2012 and 2014. The NEDIS was created in 2003, and 142 EDs throughout South Korea have participated since 2011, consisting of 21 level I regional centers, 2 of 2 specialized emergency centers, 111 of 115 level II regional emergency centers, and 8 of 323 level III regional emergency rooms [11]. Patient information was transferred automatically in real-time from each ED to the National Emergency Medical Center. These data were qualified as accurate and results were reported annually to the Ministry of Health and Welfare [11]. We used the resident population data of the Ministry of Government Administration and Home Affairs, accessible from the homepage of Korean Statistical Information Service (http://kosis.kr). The resident population data represented registered residents only, and the data were presented in 5-year age groups for every month of the calendar year. This data excluded foreign residents. The Institutional Review Boards (IRB) of Asan Medical Center approved the study before its commencement and waived the requirement for informed consent due to the retrospective nature of the study and the use of an anonymised dataset (IRB approval number 2016–0570). This study was carried out in accordance with the approved guidelines.

All adult patients (≥20 years old) who presented to the ED due to traffic accident injuries between January 2012 and December 2014 were identified. Among them, we included riders and passengers whose injuries were associated with bicycle use. Pedestrians hit by bicycles were excluded. According to the Advanced Trauma Life Support (ATLS) guideline, death due to injury occurs in one of three time periods following trauma: 1) within seconds to minutes; 2) within minutes to several hours; 3) within hours to days or weeks due to sepsis or multiple organ failure. Patients at risk of dying in the second period can often be saved by rapid assessment and resuscitation [12]. In particular, the second peak occurs within minutes to several hours following injury and these patients can be saved by rapid assessment and resuscitation [12]. We intended to include such patients having a high risk of death in the serious outcome group. Specifically, serious outcomes were defined as death in the ED, need for an emergency operation, or admission to the ICU.

### Data collection

Demographic and clinical data collected from the NEDIS dataset included age, sex, ED presentation date, geographic location of the ED, vital signs and consciousness level at ED presentation, use of safety equipment such as a helmet and safety gear (gloves, goggles, masks, and protectors), and disposition after ED management (discharge, transfer to other hospital, admission to general ward or ICU, operation room for emergency operation, death, and others). The consciousness level at ED presentation was assessed using the AVPU ("alert, voice, pain, unresponsive") scale, and patients with anything less than A were considered as having a decreased level of consciousness. In South Korea, emergency medical service (EMS) providers are encouraged to bring patients to the nearest ED, and are not permitted to cross the administrative district. We assumed the injury location was the ED location with respect to urban or rural area designation. NEDIS includes basic questions about safety equipment (including use of helmets and other protective gear) for all trauma patients, which are recorded as "yes" or "no" for each item. In addition, we acknowledged the NEDIS data do not provide specific information regarding injury mechanisms (bicycle—motor vehicle crashes, bicycle—bicycle crashes, or falls) and circumstances (road type and actual location).

### Statistical analysis

Trend data are usually reported in three-month or six-month intervals. Considering the number of subgroups, the study period was divided by an interval of 6 months. The frequencies of the demographic and clinical characteristics of each period were calculated. The temporal trends of those variables were analysed by the Mantel-Haenzsel test for trend. Logistic regression analysis was used to calculate the age- and sex-adjusted OR with 95% CI of safety helmet use for serious outcomes. Two-sided *P* values < 0.05 were considered statistically significant. All statistical analyses were performed using SPSS version 21.0 (SPSS Inc., Chicago, IL, USA).

## Results

Among 529,278 traffic-related trauma cases over a 3-year period, 58,352 (11.0%) were identified as bicycle-related injuries (Table 1). The majority of patients (80.6%) were less than 65 years of age, either 30 to 64 years old (59.0%) or 20 to 29 years (21.6%). The 100,000 population-based rate of bicycle injury by age group was highest in the 65 to 74 year olds group between 2012 and 2013, while highest in the 20 to 29 year olds group in 2014. Approximately three-quarters of the patients were male (75.2%), and the ratio of rural to urban injury locations was approximately 1:1.

**Table 1:** Demographic characteristics of patients who visited the emergency department with bicycle injuries in South Korea, 2012–2014.

Variable	First half of 2012	Second half of 2012	First half of 2013	Second half of 2013	First half of 2014	Second half of 2014	Total	P-value
Number of bicycle injury cases	7894	7430	9712	10391	10043	12882	58352	
Number of traffic-related trauma cases	77525	72842	93577	89919	89678	105737	529278	
Percent of bicycle injury cases in traffic-related trauma cases	10.2%	10.2%	10.4%	11.6%	11.2%	12.2%	11.0%	
Age, years								<0.001
20–29	1629 (20.6%)	1440 (19.4%)	2127 (21.9%)	2116 (20.4%)	2536 (23.5%)	2957 (23.0%)	12625 (21.6%)	
30–39	1087 (13.8%)	1007 (13.6%)	1393 (14.3%)	1449 (13.9%)	1435 (14.3%)	1862 (14.5%)	8233 (14.1%)	
40–49	1386 (17.6%)	1290 (17.4%)	1619 (16.7%)	1889 (18.2%)	1636 (16.3%)	2226 (17.3%)	10046 (17.2%)	
50–59	1647 (20.9%)	1584 (21.3%)	1956 (20.1%)	2142 (20.6%)	2007 (20.0%)	2602 (20.2%)	11938 (20.5%)	
60–69	1176 (14.9%)	1104 (14.9%)	1416 (14.6%)	1405 (13.5%)	1304 (13.0%)	1629 (12.6%)	8034 (13.8%)	
70–79	798 (10.1%)	826 (11.1%)	976 (10.0%)	1123 (10.8%)	1063 (10.6%)	1290 (10.0%)	6076 (10.4%)	
≥80	171 (2.2%)	179 (2.4%)	225 (2.3%)	267 (2.6%)	242 (2.4%)	316 (2.5%)	1400 (2.4%)	
Age group, years								<0.001
20–29	1629 (20.6%)	1440 (19.4%)	2127 (21.9%)	2116 (20.4%)	2536 (23.5%)	2957 (23.0%)	12625 (21.6%)	
30–64	4712 (59.7%)	4444 (59.8%)	5718 (58.9%)	6235 (60.0%)	5768 (57.4%)	7576 (58.8%)	34454 (59.0%)	
65–74	1086 (13.8%)	1051 (14.1%)	1281 (13.2%)	1332 (12.8%)	1223 (12.2%)	1513 (11.7%)	7486 (12.8%)	
≥75	467 (5.9%)	495 (6.7%)	586 (6.0%)	708 (6.8%)	696 (6.9%)	836 (6.5%)	3788 (6.5%)	
100,000 population-based rate by age group								
20–29	24.5	21.7	32.2	32.1	38.3	44.5	31.8	
30–64	17.4	16.3	20.9	22.7	20.9	27.5	21.0	
65–74	30.0	28.6	34.2	35.2	31.8	39.0	33.2	
≥75	20.8	21.5	24.4	28.8	27.1	31.7	25.9	
Sex								0.680
Male	5898 (74.7%)	5579 (75.1%)	7536 (75.7%)	7879 (75.8%)	7557 (75.2%)	9609 (74.6%)	43878 (75.2%)	
Female	1996 (25.3%)	1851 (24.9%)	2356 (24.3%)	2512 (24.2%)	2486 (24.8%)	3273 (25.4%)	14474 (24.8%)	
Location								0.178
Rural area	3693 (46.8%)	3572 (48.1%)	4659 (48.0%)	4922 (47.4%)	4898 (48.8%)	6155 (47.8%)	27899 (47.8%)	
Urban area	4201 (53.2%)	3858 (51.9%)	5053 (52.0%)	5469 (52.6%)	5145 (51.2%)	6727 (52.2%)	30453 (52.2%)	

P-value calculated by linear-by-linear association.

**Fig 1:**
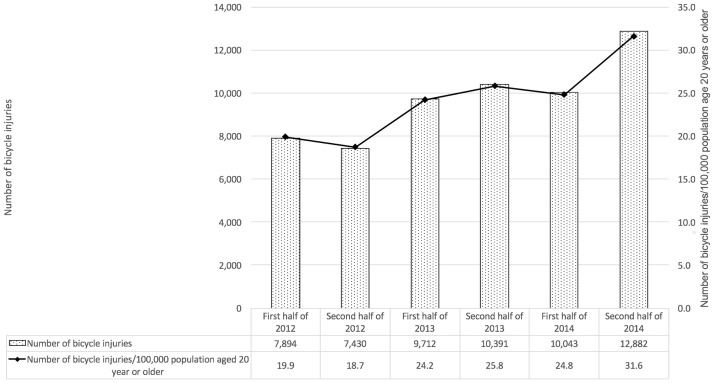
Number and proportion of bicycle-related injuries during the study period.

The number of bicycle-related injuries increased by 63.2% from 7,894 in the first half of 2012 to 12,882 in the second half of 2014 (Fig 1). The proportion of bicycle injuries among traffic-related trauma injuries increased by 19.6%, from 10.2% in the first half of 2012 to 12.2% in the second half of 2014, and the number of bicycle-related injuries presenting in EDs rose from 19.9 to 31.6 per 100,000 population aged 20 years or older during the same time period. The incidence of bicycle-related injuries also changed significantly with age; the proportions of patients < 30 years and ≥75 years increased over the study period (p < 0.001). In contrast, the male-to-female ratio and urban-to-rural ratio did not change during the study period.

Table 2 presents the clinical characteristics and outcomes of the study patients. The proportion of patients with a low systolic blood pressure (≤90 mmHg) and those with decreased level of consciousness decreased from 1.7% to 1.3% (p < 0.001) and from 2.9% to 2.3% (p < 0.001), respectively. In addition, the overall proportion of serious outcomes decreased from 5.0% to 4.2% during the study period (p < 0.001). The majority of patients (72.8%) were discharged from the ED after treatment. Also, the rate of helmet use increased by 43.0% from between the first half of 2012 (14.2%) and the second half of 2014 (20.3%) (p < 0.001), but remained low at 16.8% over the entire study period.

**Table 2:** Clinical characteristic and outcomes of patients who visited EDs due to bicycle injuries in South Korea, 2012–2014.

Variable	First half of 2012	Second half of 2012	First half of 2013	Second half of 2013	First half of 2014	Second half of 2014	Total	P-value
Status at ED presentation								0.017
Stable	7762 (98.3%)	7321 (98.5%)	9607 (98.9%)	10248 (98.6%)	9928 (98.9%)	12718 (98.7%)	57584 (98.7%)	
Unstable	132 (1.7%)	109 (1.5%)	105 (1.1%)	143 (1.4%)	115 (1.1%)	164 (1.3%)	768 (1.3%)	
Consciousness at ED presentation								<0.001
Alert	7667 (97.1%)	7195 (96.8%)	9475 (97.6%)	10107 (97.3%)	9791 (97.5%)	12592 (97.7%)	56827 (97.4%)	
Decreased level of consciousness	227 (2.9%)	235 (3.2%)	237 (2.4%)	284 (2.7%)	252 (2.5%)	290 (2.3%)	1525 (2.6%)	
Safety equipment	1151 (14.6%)	1223 (16.5%)	1587 (16.3%)	1928 (18.6%)	1827 (18.2%)	2656 (20.6%)	10372 (17.8%)	<0.001
Helmet use	1122 (14.2%)	1171 (15.8%)	1539 (15.8%)	1874 (18.0%)	1790 (17.8%)	2616 (20.3%)	10112 (17.3%)	
Other gear use	29 (0.4%)	52 (0.7%)	48 (0.5%)	54 (0.5%)	37 (0.4%)	40 (0.3%)	260 (0.4%)	
None	6743 (85.4%)	6207 (83.5%)	8125 (83.7%)	8463 (81.4%)	8216 (81.8%)	10226 (79.4%)	47980 (82.8%)	
Disposition at ED								<0.001
Discharge	5699 (72.2%)	5230 (70.4%)	6916 (71.2%)	7575 (72.9%)	7488 (74.6%)	9592 (74.5%)	42500 (72.8%)	
Transfer to other hospital	241 (3.1%)	241 (3.2%)	282 (2.9%)	297 (2.9%)	252 (2.5%)	329 (2.6%)	1642 (2.8%)	
Admission	1752 (22.2%)	1782 (23.9%)	2293 (23.5%)	2325 (22.4%)	2102 (21.0%)	2735 (21.2%)	12989 (22.3%)	
*General ward*	1540 (19.5%)	1512 (20.3%)	2027 (20.9%)	2017 (19.4%)	1874 (18.7%)	2397 (18.6%)	11367 (19.5%)	
*ICU admission*	212 (2.7%)	270 (3.6%)	266 (2.6%)	308 (3.0%)	228 (2.3%)	338 (2.6%)	1622 (2.8%)	
Emergency operation	142 (1.8%)	122 (1.6%)	159 (1.6%)	140 (1.3%)	139 (1.4%)	169 (1.3%)	871 (1.5%)	
Death in ED	41 (0.5%)	35 (0.5%)	40 (0.4%)	37 (0.4%)	39 (0.4%)	35 (0.3%)	227 (0.4%)	
Others	19 (0.2%)	20 (0.3%)	22 (0.2%)	17 (0.2%)	23 (0.2%)	22 (0.2%)	123 (0.2%)	
Serious outcome								<0.001
Yes	395 (5.0%)	427 (5.7%)	465 (4.8%)	485 (4.7%)	406 (4.0%)	542 (4.2%)	2720 (4.7%)	
No	7499 (95.0%)	7003 (94.3%)	9247 (95.2%)	9906 (95.3%)	9637 (96.0%)	12340 (95.8%)	55632 (95.3%)	

P-value calculated by linear-by-linear association.

ED, emergency department; ICU, intensive care unit.

**Fig 2:**
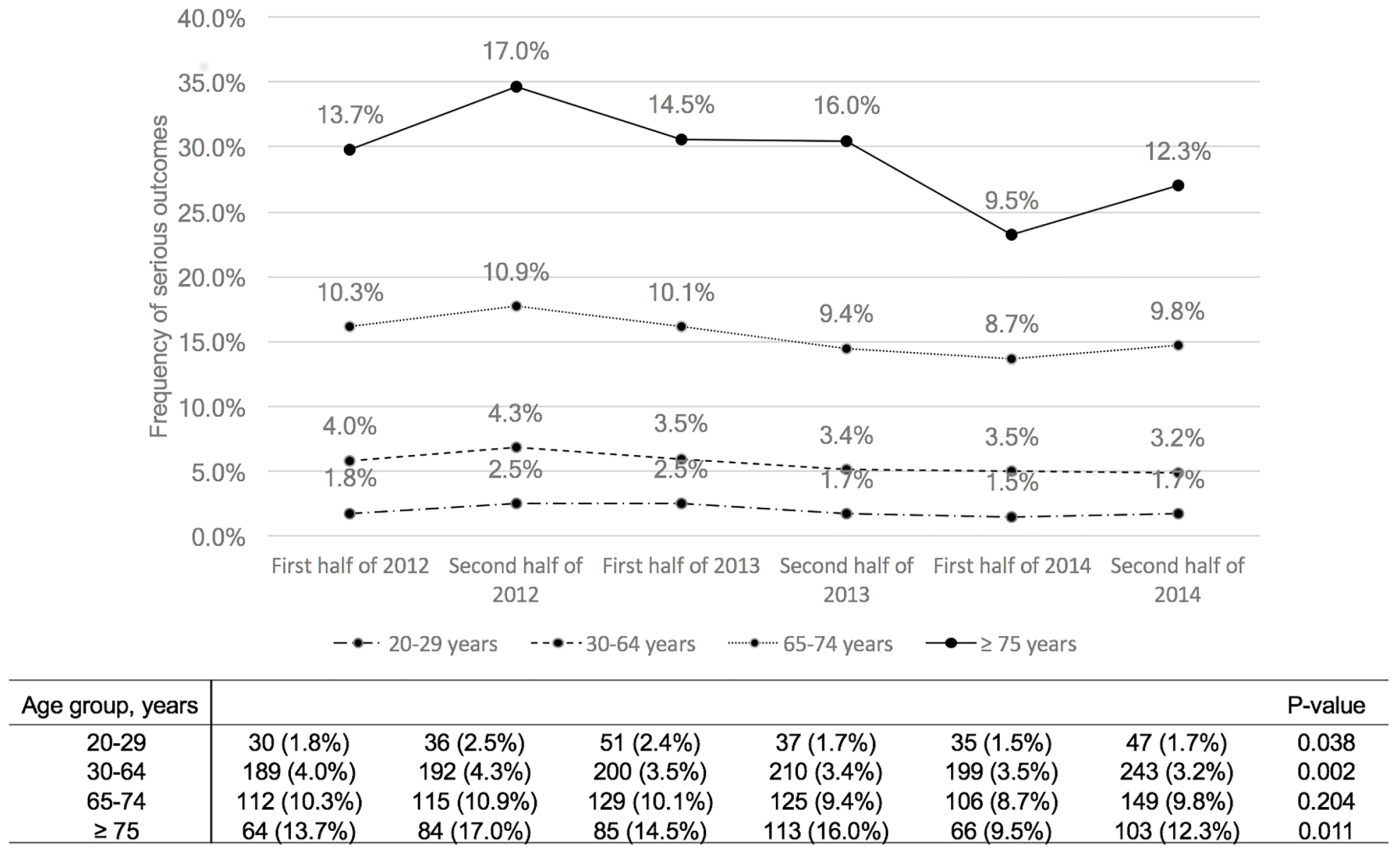
Frequency of serious outcomes in patients with bicycle-related injuries by age group.

**Fig 3:**
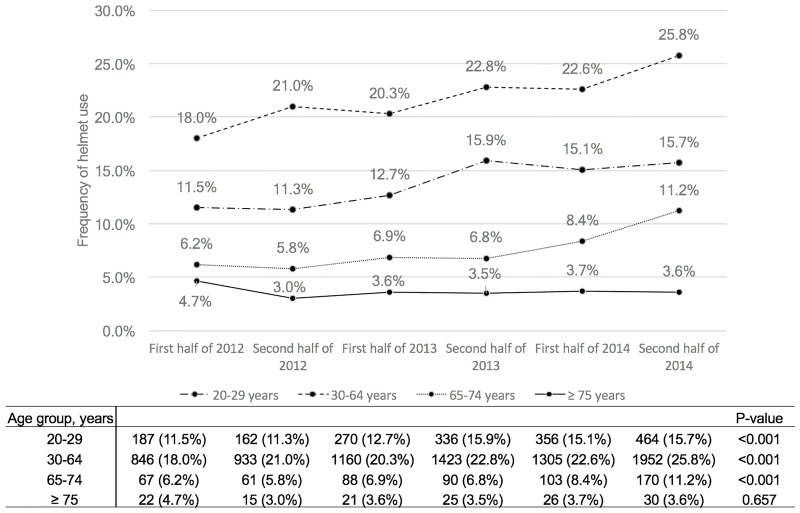
Frequency of helmet use in patients with bicycle-related injuries by age group.

We also evaluated the association between helmet use and serious injury outcomes by age group. Fig 2 showed that the serious outcome incidence was higher in the elderly (65–74 years) and older elderly (≥75 years) groups; in contrast to all of the other age groups, the elderly (65–74 years) group had a consistent proportion of serious outcomes during the study period (9.8%; p = 0.204). Fig 3 shows the helmet use rate by age group, which increased gradually in all age groups except for the older elderly (≥75 years) group (p < 0.001). The helmet use rate was the lowest in the older elderly (≥75 years) group (3.6%) and did not change significantly during the study period (p = 0.657). Not using a helmet was significantly associated with serious outcomes, controlling for age and sex (odds ratio [OR], 1.811; 95% confidence interval [CI], 1.576–2.082) (Table 3 and S1 Table).

**Table 3:** Adjusted odds ratios for serious outcomes in patients with bicycle-related injuries.

	Odds Ratio	95% Confidence Interval	P-value
No use of helmet	1.811	1.576–2.082	< 0.001
Age group			
20–29	Reference		
30–39	1.058	0.862–1.298	0.591
40–49	1.645	1.382–1.959	< 0.001
50–59	2.562	2.192–2.995	< 0.001
60–69	4.276	3.669–4.984	< 0.001
70–79	6.350	5.457–7.388	< 0.001
≥80	7.974	6.544–9.716	< 0.001
Female	0.710	0.640–0.787	< 0.001

## Discussion

This population-based study evaluated temporal epidemiologic changes in bicycle-related injuries presenting to EDs in South Korea between 2012 and 2014. We found that the incidence of bicycle-related injuries increased gradually in all groups but that the proportion of serious outcome decreased during the study period in all but the elderly group (65–74 years). The rate of helmet use also increased continuously, but it remained low. Notably, the rate of helmet use was lowest (3.6%) among older elderly patients (≥75 years) and did not increase during the study period, and older elderly patients showed the highest frequency of serious outcomes.

We found an increasing incidence of bicycle-related injuries in South Korea, which might be a result of government policies to promote bicycling and the popularity of bicycling as a leisure activity, which increases the likelihood of bicycle-related injuries. In our study, 80.6% of the injured patients were < 65 years of age, and the proportion of patients in both extreme age groups (20–29 years and ≥75 years) increased over the study period. These findings indicate that bicycle-related injuries could create a large financial burden on individuals and healthcare systems, as well as lead to a loss of human resources. From this point-of-view, social and national attention is required to prevent injury. Continuous public education and campaigns for bicycle riders about safely sharing the roads and the effectiveness of helmets in mitigating serious head injury are necessary. Additional legislation about the penalty for violating the Road Traffic Act should be considered.

Our results are consistent with those of previous epidemiologic studies in terms of demographic features, such as age and sex. Approximately one-fifth (19.3%) of the injured patients were elderly, and male sex was predominant (75.2%) [5, 13, 14]. Although we did not include pediatric and adolescent patients (<20 years), many previous studies demonstrated that pediatric and elderly patients were vulnerable to bicycle injuries and prone to fatal outcomes [5, 7, 8, 13, 15–17]. In our study, the overall proportion of serious outcomes decreased during the study period, but elderly patients consistently had a higher frequency of serious outcomes. Therefore, it is important to design and implement age group-focused injury prevention strategies and refer to the injury prevention strategies of other developed countries during this transition period with a relatively low-level of bicycle-friendly infrastructure and safety awareness.

As the proportion of serious outcomes decreased, that of safety equipment use gradually increased. Safety helmet use is a simple but well-known strategy for preventing injury [10, 17–19]. A recent systemic review of data from forty studies found that helmets are clearly effective in a crash without associated increases in other injuries, with odds reductions of 51% for head injury, 69% for serious head injury, 33% for face injury and 65% for fatal head injury, which support the use of strategies to increase the uptake of bicycle helmets [10]. In our study, the majority of older elderly patients (≥75 years) did not wear a helmet, which presented a significant risk factor for a serious outcome (OR, 1.811; 95% CI, 1.576–2.082). This finding reflects a difference in the perceived safety risk associated with bicycling between the young and the elderly and the need for age-specific education.

Previous studies reported that poor road surface quality, alcohol consumption, and lack of cycling experience were also contributing factors for bicycle injuries [8, 20]. Therefore, the mainstay of injury prevention approaches is public education and legislation, as well as the design and implementation of a bicycle-specific infrastructure [7, 8, 13, 18, 21]. Controversy over whether bicycle helmet legislation deters bicycle riders persists despite a lack of strong evidence; however, studies with improved designs have indicated that bicycle helmet legislation is a non-factor, but rather a lack of infrastructure or concern for safety are the main deterrents of riding a bicycle [22, 23]. Many studies demonstrated the implementation of bicycle helmet legislation has been shown to greatly reduce bicycle-related traumatic brain injuries [18, 19, 21, 24, 25]. In South Korea, bicycle helmet use for children has been specified in Article 50 (Matters to be Observed by Specific Drivers) of the Road Traffic Act since 2010. However, there is no legislation on helmet use for adults. The current study’s results can improve and strengthen South Korea’s bicycle-related injury prevention policies.

This study had several limitations. The data were collected from the NEDIS dataset, which does not include data from low-level EDs. Given that our results did not represent all bicycle-related injuries, the real incidence may be underestimated, especially for minor injuries or very severe injuries of which patients died at the scene. However, NEDIS data presumably included most of the clinically significant patients who needed treatment for their bicycle injuries. In addition, the NEDIS data do not provide specific information regarding injury mechanisms (bicycle-motor vehicle crashes, bicycle-bicycle crashes, or falls), circumstances (road type and actual location), and the treatment or diagnostic workup performed at the ED. However, our NEDIS dataset included EMS information and detailed disposition after ED treatment, which was not provided to other national registries.

In conclusion, the incidence of bicycling-related injuries has been increasing, and is expected to continue to do so, in South Korea. Although there was a decline in the incidence of serious outcomes among bicycle injuries, possibly related to the increased use of safety equipment, the total number of bicycle-related injuries, including those with serious outcomes, grew between 2012 and 2014, reflecting an overall increased socioeconomic burden of injuries. Injury prevention strategies, such as public education campaigns to promote safety equipment use and safe riding practices, especially amongst the elderly, as well as the implementation of infrastructure to mitigate the risk of injury can be effective tools in reducing the injury burden while promoting the health and environmental benefits of bicycling.

## Supporting information

S1 Table The stratified counts by age group, sex, and outcome level. (DOCX)
