# Supplementary material for: Trends in the incidence and outcomes of bicycle-related injury in the emergency department: A nationwide population-based study in South Korea, 2012-2014
Source: PLoS One. 2017 Jul 13;12(7):e0181362. doi: 10.1371/journal.pone.0181362 (PMC5509316; doi:10.1371/journal.pone.0181362)
Supplement: S1 Table — (DOCX) [file pone.0181362.s001.docx]

**Supplement 1. The stratified counts by age group, sex, and outcome level**

|  |  |  | Outcome | | Total |
| --- | --- | --- | --- | --- | --- |
| Age group |  |  | Non-serious outcome | Serious outcome |  |
| 20-29 | Sex | Female | 3125 | 47 | 3172 |
|  |  | Male | 9264 | 189 | 9453 |
| 30-39 | Sex | Female | 1890 | 14 | 1904 |
|  |  | Male | 6188 | 141 | 6329 |
| 40-49 | Sex | Female | 2704 | 67 | 2771 |
|  |  | Male | 7054 | 221 | 7275 |
| 50-59 | Sex | Female | 3535 | 122 | 3657 |
|  |  | Male | 7876 | 405 | 8281 |
| 60-69 | Sex | Female | 1959 | 141 | 2100 |
|  |  | Male | 5467 | 467 | 5934 |
| 70-79 | Sex | Female | 731 | 80 | 811 |
|  |  | Male | 4643 | 622 | 5265 |
| ≥80 | Sex | Female | 56 | 3 | 59 |
|  |  | Male | 1140 | 201 | 1341 |
| Total | Sex | Female | 14000 | 474 | 14474 |
|  |  | Male | 41632 | 2246 | 43878 |
